# Characterisation and protein expression profiling of annexins in colorectal cancer

**DOI:** 10.1038/sj.bjc.6604128

**Published:** 2007-12-11

**Authors:** R Duncan, B Carpenter, L C Main, C Telfer, G I Murray

**Affiliations:** 1 Department of Pathology, University of Aberdeen, Aberdeen, UK; 2 Auvation Ltd, Crombie Lodge, Aberdeen Science Park, Balgownie Drive, Aberdeen, UK

**Keywords:** annexin, immunohistochemistry, proteomics, prognosis, tissue microarray

## Abstract

The annexins are family of calcium-regulated phospholipid-binding proteins with diverse roles in cell biology. Individual annexins have been implicated in tumour development and progression, and in this investigation a range of annexins have been studied in colorectal cancer. Annexins A1, A2, A4 and A11 were identified by comparative proteomic analysis to be overexpressed in colorectal cancer. Annexins A1, A2, A4 and A11 were further studied by immunohistochemistry with a colorectal cancer tissue microarray containing primary and metastatic colorectal cancer and also normal colon. There was significant increase in expression in annexins A1 (*P*=0.01), A2 (*P*<0.001), A4 (*P*<0.001) and A11 (*P*<0.001) in primary tumours compared with normal colon. There was increasing expression of annexins A2 (*P*=0.001), A4 (*P*=0.03) and A11 (*P*=0.006) with increasing tumour stage. An annexin expression profile was identified by *k*-means cluster analysis, and the annexin profile was associated with tumour stage (*P*=0.01) and also patient survival. Patients in annexin cluster group 1 (low annexin expression) had a better survival (log rank=5.33, *P*=0.02) than patients in cluster group 2 (high annexins A4 and A11 expression). In conclusion, this study has shown that individual annexins are present in colorectal cancer, specific annexins are overexpressed in colorectal cancer and the annexin expression profile is associated with survival.

The annexins are a multigene family of calcium-regulated phospholipid-binding proteins (Gerke and Moss, 2003; Gerke *et al*, 2005). The annexins are classified into five groups (A–E), and within each of these groups, individual annexins are identified numerically. Annexins in group A are human annexins, with group B referring to animal annexins without human orthologs, group C to fungi and moulds, group D to plants and group E to protists (Liemann and Huber, 1997; Rand, 2000; Hayes and Moss, 2004; Rescher and Gerke, 2004; Lim and Pervaiz, 2007). The characteristic annexin structural motif is a 70-amino-acid repeat, called the annexin repeat. Four annexin repeats packed into an *α*-helical disk are contained within the C-terminal polypeptide core (Gerke and Moss, 2003). While all annexins share this core region, the N-terminal varies widely between annexins, and it is this diversity of N-terminal amino-acid sequence that gives the individual annexins their functional differences and biological activities (Gerke and Moss, 2003; Gerke *et al*, 2005). There are 12 human annexin subfamilies (A1–A11 and A13) that have been found to have various intra- and extracellular roles in a range of cellular processes such as cell signalling, ion transport, cell division and apoptosis (Gerke and Moss, 2003; Gerke *et al*, 2005).

All annexins share an ability to bind to negatively charged phospholipid membranes in a calcium-dependent manner. This property is found within the annexin core motif where the calcium- and membrane-binding sites are located. Annexins bind to the cytosolic surface of the plasma membrane and to organelle membranes such as the Golgi apparatus. This binding can be reversed by the removal of calcium, freeing the annexin from the phospholipid membrane. However, the functional significance of their reversible membrane-binding ability remains unknown in many annexins, although in some it is thought to be important for vesicle aggregation and membrane organisation (Liemann and Huber, 1997; Rand, 2000; Rescher and Gerke, 2004; Lim and Pervaiz, 2007). Although all annexins share this binding property, there is variation in calcium sensitivity and phospholipid specificity between individual annexins. For example, within one cell there can be differences in the distribution of annexins, with annexin A1 having an endosomal localisation, A2 to be found in cytosol and A4 being associated with the plasma membrane (Liemann and Huber, 1997).

Some annexins are capable of calcium-independent binding and several have roles in vesicle aggregation. Annexins A1, A2 and A11 function in cooperation with other calcium-binding proteins to form complexes while annexins A1, A2 and A5 interact with cytoskeletal proteins. Many annexins are involved in exocytic and endocytic pathways and some have roles in ion channel regulation (Gerke and Moss, 2003). Extracellularly, annexin A1 has a role in controlling the inflammatory response while annexin A2 is present on the external surface of endothelial cells, where it may act as a receptor for ligands, including plasminogen and tissue plasminogen activator (Diaz *et al*, 2004; Roda *et al*, 2006). Extracellular annexin A5 is thought to be involved in the anticoagulation process (Rand, 2000; Hayes and Moss, 2004).

Annexins have been implicated in several disease processes, including inflammation and neoplasia (Rand, 2000). Alterations in the expression of individual annexins have been associated with tumorigenesis in several types of tumour. Loss of annexin A1 has been found to be an early event in oesophageal squamous cell carcinoma, and it may function as a tumour suppressor in the development of this type of tumour (Paweletz *et al*, 2000). Expression of annexin A1 has also been associated with poorly differentiated oesophageal tumours of higher tumour stage (Hu *et al*, 2004). Together, these studies suggest that dysregulation of annexin A1 loss is important in oesophageal tumour development and progression. Other types of cancer in which the expression of annexin A1 has been shown to be altered include pancreatic adenocarcinoma (Bai *et al*, 2004), where it was found to show increased expression in the majority of tumours, renal cell carcinoma (Zimmermann *et al*, 2007), prostate adenocarcinoma (Patton *et al*, 2005), breast cancer (Shen *et al*, 2006) and B-cell non-Hodgkin's lymphoma (Vishwanatha *et al*, 2004).

Other annexins have also been implicated in tumorigenesis. Overexpression of annexin A2 has been found in renal cell cancer, where it is associated with tumour stage (Zimmermann *et al*, 2004a), invasive breast cancer (Sharma *et al*, 2006) and sarcomas, including both soft tissue sarcomas (Syed *et al*, 2007) and osteosarcomas (Gillette *et al*, 2004). There is increased expression of annexin A4 in renal clear cell carcinoma (Zimmermann *et al*, 2004b). In prostate cancer, decreased expression of annexin A4 has been shown to correlate with worsen pathological stage (Xin *et al*, 2003), and loss of annexin A7 has been associated with metastatic and local recurrences of hormone refractory prostate cancer (Srivastava *et al*, 2001).

However, the annexins have received no significant study in colorectal cancer and in this study we used comparative proteomic analysis to identify proteins that are overexpressed in colorectal cancer, compared with morphologically normal colorectal mucosa. The annexins A1, A2, A4 and A11 were identified as four such proteins. In order to further define the roles played by these proteins in colorectal neoplasia, their expression and cellular localisation was studied by immunohistochemistry in a large series of colorectal cancers represented within a colorectal cancer tissue microarray.

## MATERIALS AND METHODS

### Proteomics

Two-dimensional gel electrophoresis and matrix-assisted laser desorption ionisation time of flight mass spectrometry (MALDI-TOF MS) on normal colon and colorectal cancer were performed as previously described (Lawrie *et al*, 2004; Dundas *et al*, 2005; Coghlin *et al*, 2006). Proteins were solubilised from Dukes C adenocarcinoma tissue samples and patient-matched morphologically normal colorectal mucosa (*n*=10 pairs of tumour and normal samples). Two-dimensional gel electrophoresis was performed using 3–10 pI immobilon strips with proteins being separated according to charge, and subsequently molecular weight. Following completion of the electrophoresis, gels were stained with Coomassie blue to visualise protein spots. The spots were excised from the gels and peptide mass mapping was performed using a PerSeptive Biosystems Voyager-DE STR mass spectrometer. The masses of the tryptic fragments were determined and entered into the MS-Fit database-searching program (http://prospector.ucsf.edu/uc sfhtml14.0/msfit.htm). The database was restricted to searching for human proteins, but no restrictions were placed on either molecular weight or isoelectric point. To ensure that proteins were accurately identified, a significant difference in statistical score (MOWSE score) between proteins ranked first and second in the results had to be obtained.

### Antibodies

A polyclonal antibody to annexin A4 was produced in our own laboratory. An immunising peptide was designed, which took into consideration both antigenicity and hydrophilicity. Furthermore, a blast search was used to determine that the peptide was unique to annexin A4 (SVLAYRNTA corresponding to amino acids 39–47 of annexin A4), thus ensuring no crossreactivity with other proteins. The annexin IV peptide conjugated to ovalbumin was used to immunise rabbits subsequently followed with four boosters, with a protocol we have previously used for the development of other antibodies (Kumarakulasingham *et al*, 2005). Nine weeks after the initial booster, rabbits were bled and serum was tested by ELISA using the peptide as an immunogen. The sera that showed the highest antibody titre were also tested by immunoblotting using whole-cell lysates. Immunoblotting showed that the antibody recognised a protein at the expected molecular size for annexin A4.

Monoclonal antibodies to annexins A1, A2, A7 and A11 were bought from BD Biosciences (Oxford, UK).

### Tumour samples and tissue microarray construction

All cases were selected from the Aberdeen colorectal tumour bank. In total, 268 patients were involved in this study; in each case, a diagnosis of primary colorectal cancer had been made, and the patients had undergone elective surgery for primary colorectal cancer, in Aberdeen, between 1994 and 2003. The Aberdeen colorectal tumour bank is linked to a comprehensive set of clinicopathological data, including age, gender, site of primary tumour, degree of tumour differentiation and tumour stage. The data for the patients included in this study are detailed in Table 1. Complete follow-up ranging from 0 to 144 months was available for all patients, and the mean patient survival was 91 months. At the time of censoring the data, there had been 106 (39.6%) deaths in the patient group. The tumour samples were submitted to the Department of Pathology, University of Aberdeen for diagnosis. The tumour excision specimens were fixed in formalin, representative blocks were embedded in wax and sections were stained with haematoxylin and eosin. Permission for this study was obtained from the Grampian Research Ethics Committee.

A colorectal cancer tissue microarray was constructed as described (Dundas *et al*, 2005; Kumarakulasingham *et al*, 2005). The tumours within the array were representative of the distribution of anatomical locations and the Dukes stages found in colorectal cancers within this population. The tissue microarray contained primary colorectal cancer (Dukes A=53, Dukes B=104 and Dukes C=111). In addition, it contained lymph node metastases and morphologically normal colonic mucosal samples. The lymph node metastases were from the corresponding Dukes C cases (*n*=111). Each normal sample (*n*=52) was acquired from at least 10 cm distant from the tumour as previously described (Kumarakulasingham *et al*, 2005). Using a steel Menghini needle, a representative 1.6 mm core of tissue was taken from each donor block and arrayed into the recipient wax block. In order to check the histopathological diagnosis and the adequacy of tissue sampling, a section from each microarray was stained with haematoxylin and eosin and examined by light microscopy.

### Immunohistochemistry

Annexin immunohistochemistry was carried out using a Dako autostainer (Dako, Ely, UK) as previously described (Dundas *et al*, 2005; Coghlin *et al*, 2006). Sections (4 *μ*m) of the tissue microarray were dewaxed, rehydrated and an antigen retrieval step performed when required. The antigen retrieval step consisted of microwaving the sections in 0.01 M citrate buffer at pH 6.0 for 20 min in an 800 W microwave oven operated at full power. The sections were then allowed to cool to room temperature. Primary antibody appropriately diluted (Table 2) in antibody diluent (Dako) was applied for 60 min at room temperature, washed with buffer (Dako) followed by peroxidase blocking for 5 min (Dako), followed by a single 2-min buffer wash. Prediluted peroxidase polymer-labelled goat anti-mouse/rabbit secondary antibody (Envision™, Dako) was applied for 30 min at room temperature, followed by further washing with buffer to remove unbound antibody. Sites of peroxidase activity were then demonstrated with diaminobenzidine as the chromogen applied for three successive 5 min periods. Finally, sections were washed in water, lightly counterstained with haematoxylin, dehydrated and mounted. Omitting the primary antibody from the immunohistochemical procedure and replacing it with antibody diluent or non-immune rabbit serum acted as negative controls.

The sections were evaluated by light microscopic examination, and cellular localisation and intensity (negative=0, weak=1, moderate=2, strong=3) of immunostaining in each section were assessed by two observers (RD and GIM).

### Statistical analysis

Comparison of expression of individual annexins in normal colon, colon cancer and lymph node metastasis was performed with the Mann–Whitney *U*-test. The chi-square (*χ*^2^) test was used to compare annexin expression with tumour stage while the annexin expression profile was determined by *k*-means cluster analysis. The relationship of patient survival and annexin expression was determined using the method of Kaplan–Meier and the log-rank test. Cox-multivariate analysis was used to determine the relative significance of individual clinicopathological factors, annexin expression and patient survival. All the statistical analyses were performed using SPSS v15 for Windows XP™ (SPSS UK Ltd, Woking, UK).

## RESULTS

### Proteomics

**Figure 1:**
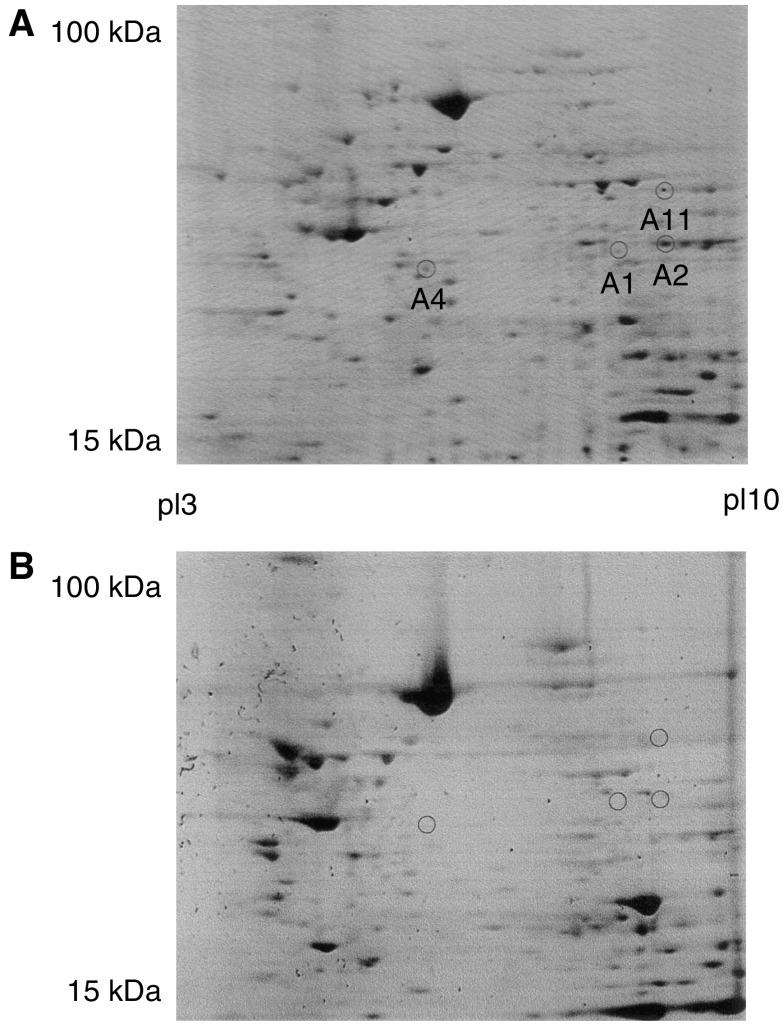
Two-dimensional gels of (**A**) colorectal cancer and (**B**) normal colon mucosa. The circles labelled A1, A2, A4 and A11 correspond to the protein spots annexins A1, A2, A4 and A11, respectively (Coomassie blue-stained two-dimensional electrophoresis gel).

Comparative proteomic analysis using two-dimensional gel electrophoresis identified certain protein spots that were represented in the colorectal cancer samples but not in the normal colorectal mucosal samples (Figure 1). Protein spots of interest were digested and the masses of the tryptic fragments were determined using MALDI-TOF MS. These masses were entered into MS-Fit, which identified annexins A1, A2, A4 and A11 with a high degree of significance. The MOWSE score (a measure of the identity of the protein) for annexin A1 was 1.68e+003, annexin A2 was 6.53e+005, annexin A4 was 1.52e+008 and annexin A11 was 6.67e+005.

### Immunohistochemistry

#### Primary colorectal cancer

**Figure 2:**
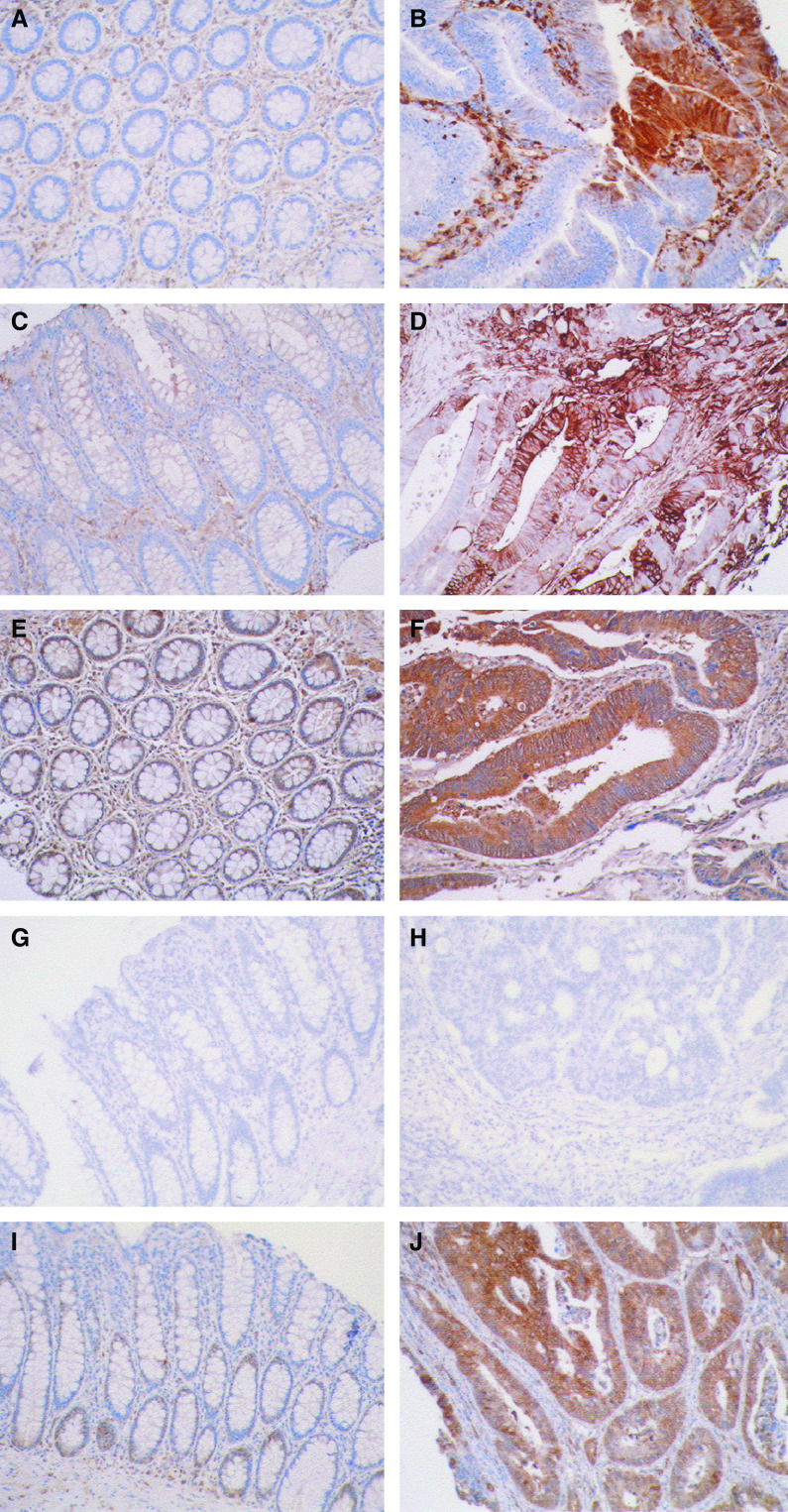
The immunohistochemical localisation of annexins in normal colon and colorectal cancer. Normal colon (**A, C, E, G** and **I**) and colorectal cancer (**B, D, F, H** and **J**). Annexin A1 (**A** and **B**), annexin A2 (**C** and **D**), annexin A4 (**E** and **F**), annexin A7 (**G** and **H**) and annexin A11 (**I** and **J**).

**Figure 3:**
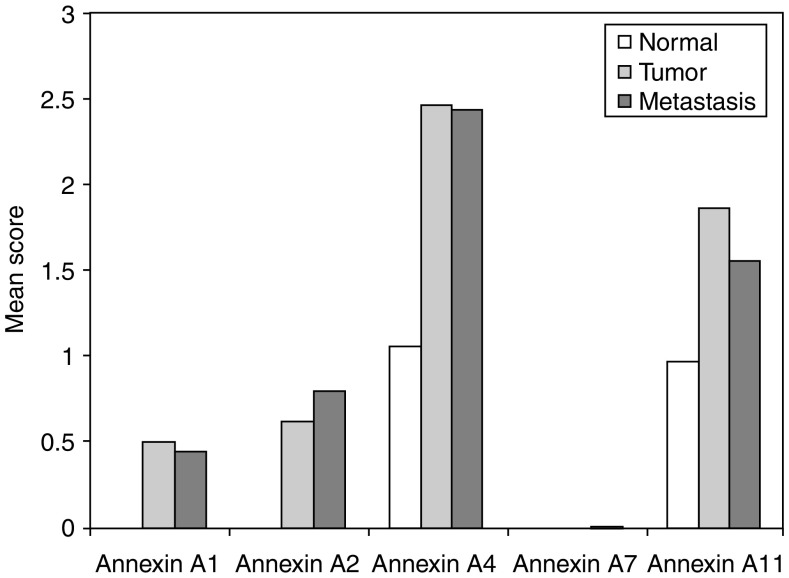
The mean intensity of expression of individual annexins in normal colon, primary colorectal cancer and lymph node metastasis.

The annexins with the exception of annexin A7 showed increased immunostaining in primary tumours in comparison to normal colon (Figures 2 and 3). Weak staining was seen for all annexins varying from 0.4 to 25.9% of tumours. Moderate and strong tumour cell staining was seen in annexins A1, A2, A4 and A11. More tumours showed strong staining for A1, A2 and A4 than showed moderate staining. The highest percentage of strong staining was seen in annexin A4, with 64.9% of tumours showing strong staining. However, for annexin A11, moderate staining was observed in 33.3% of tumours compared to 31.1% of tumours that showed strong staining (Figures 2 and 3). There was significant increase in expression in annexins A1 (*P*=0.01), A2 (*P*<0.001), A4 (*P*<0.001) and A11 (*P*<0.001) in primary tumours compared with normal colon (Figure 3). There was increased expression of annexins A2, A4 and A11 with increasing tumour stage (Table 3).

#### Lymph node metastasis

**Figure 4:**
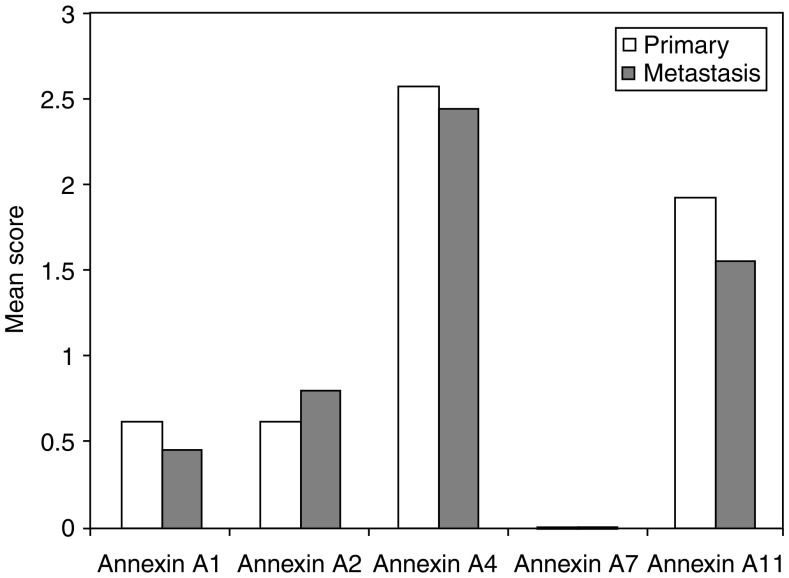
Comparison of the mean intensity of annexin expression in Dukes C primary tumours and corresponding lymph node metastasis.

The annexins except annexin A7 showed immunoreactivity in lymph node metastasis. Annexins A2 and A11 showed the greatest percentage of weak staining at 20.7 and 29%, respectively, with less tumours showing moderate and strong staining for these annexins. As in normal and primary tumours, annexin A4 showed the greatest percentage of strong staining at 62.3% of tumours. Comparing the expression of annexins in lymph node metastasis with the corresponding primary tumours showed that there was a significant decrease in expression of annexin A11 (*P*=0.01) in lymph node metastasis compared with corresponding primary colorectal cancers (Figure 4).

### Annexin expression profile and clinicopathological factors

**Figure 5:**
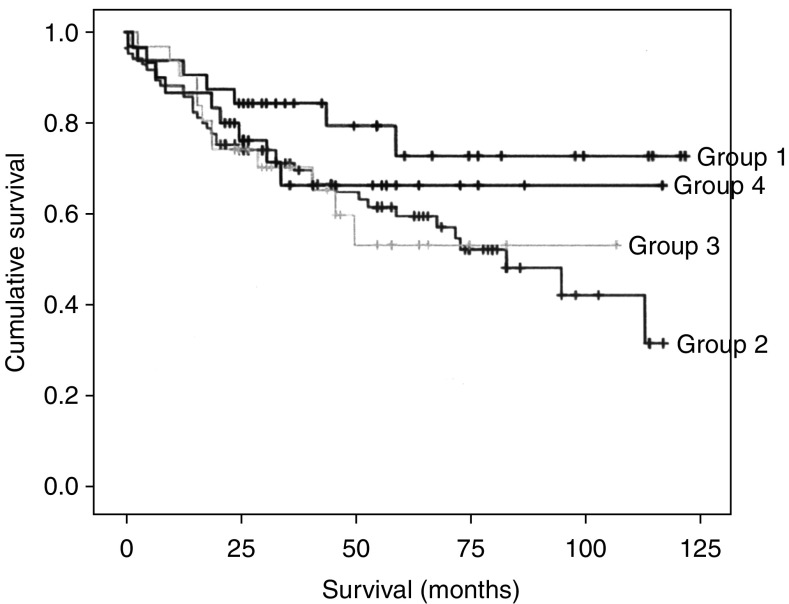
Patient survival in annexin cluster groups. There is a significant difference in survival between cluster group 1 (low annexin expression) and cluster group 2 (high annexins A2 and A11 expression, log rank=5.33; *P*=0.02).

To further dissect the role of annexin expression in colorectal cancer, the annexin expression profile was determined. To identify the relationship of the overall annexin profile within tumours, *k*-means cluster analysis was performed and this identified four clusters or groups with distinct annexin profiles (Table 4). The annexin profile was associated with Dukes stage (*χ*^2^=16.76, *P*=0.01). The annexin expression profile was also associated with survival. Patients in annexin cluster group 1 (low annexin expression) had a better survival (log rank=5.33, *P*=0.02; Figure 5) than patients in cluster group 2 (high annexin A4 and A11 expression). The mean survival in group 1 was 96 months (95% CI: 80–113 months) and in group 2 was 72 months (95% CI: 62–82 months). However, there was no relationship between the expression of individual annexins and patient survival and also the annexin profile was not an independent marker of prognosis following multivariate analysis.

## DISCUSSION

The annexins are a multigene family of calcium-dependent phospholipid-binding proteins (Gerke and Moss, 2003; Hayes and Moss, 2004; Gerke *et al*, 2005). There are 12 human annexins each of which shows a cell- and tissue-type-specific pattern of expression. Some of the annexins have been well characterised while less is known about the biology of some of the other annexins. Functions of the annexins include vesicle aggregation and ion channel regulation as well as roles in cell cycle regulation, cell signalling, cell differentiation and as extracellular receptors. Several of the annexins have been linked to the pathogenesis of a variety of disease processes, including the development and progression of several different types of cancer, and from these studies there appears to be tumour-type-specific alterations in the expression of individual annexins (Paweletz *et al*, 2000; Gerke and Moss, 2003; Xin *et al*, 2003; Bai *et al*, 2004; Gillette *et al*, 2004; Hayes and Moss, 2004; Hu *et al*, 2004; Vishwanatha *et al*, 2004; Patton *et al*, 2005; Sharma *et al*, 2006; Shen *et al*, 2006; Zimmermann *et al*, 2004a, 2004b, 2007; Syed *et al*, 2007). However, the annexin profile has not previously been investigated in individual tumour types. Furthermore, while there has been a range of studies of individual annexin expression in specific types of primary tumours, there have been no significant previous studies of annexin expression in metastasis. In addition, more recently individual annexins have been proposed as putative tumour biomarkers and potential therapeutic targets in cancer (Oh *et al*, 2004; Falsey *et al*, 2006; Wozny *et al*, 2007).

The expression and cellular localisation of annexins have received very little previous study in colorectal cancer, with only annexin A2 having been investigated (Emoto *et al*, 2001). In this study, comparative proteomic analysis utilising two-dimensional gel electrophoresis and mass spectrometry identified four annexins, namely A1, A2, A4 and A11 to be overexpressed in colorectal cancer when compared with normal colon. The expression profile of these annexins was then validated on a large cohort of well-characterised colorectal cancer samples, and the immunohistochemical component of the study was also extended to include a further annexin, annexin A7, as it has also been implicated in tumorigenesis (Srivastava *et al*, 2001). The relationship of annexin expression with clinicopathological parameters, in particular, tumour stage and overall patient survival was also investigated.

Annexins A1, A2, A4 and A11 were found to be overexpressed in primary colorectal cancer and expression of the annexins A2, A4 and A11 all increased significantly with advancing tumour stage. These findings suggest that annexins A2, A4 and A11 have an important role in the progression of colorectal cancer. In support of these findings, several studies have implicated individual annexins in tumorigenesis. Annexin A1 has been reported to show altered expression in a variety of different cancers, including oesophageal cancer (Paweletz *et al*, 2000; Hu *et al*, 2004; Wang *et al*, 2006), pancreatic cancer (Bai *et al*, 2004) and hairy cell leukaemia (Falini *et al*, 2004). In oesophageal adenocarcinoma, the tumour cell expression of annexin A1 has been associated with poor prognosis (Wang *et al*, 2006). Furthermore, annexin A1 has been found to be overexpressed in immortalised colorectal cell lines (Guzman-Aranguez *et al*, 2005). This annexin has also a potential role in tumour invasion and metastasis, as inhibition of annexin A1 using siRNA resulted in a significant reduction of cell invasion using an *in vitro* assay on an immortalised colorectal cancer cell line (Babbin *et al*, 2006).

Annexin A2 shows increased expression in several type of cancer, including renal cell cancer (Zimmermann *et al*, 2004a), breast cancer (Sharma *et al*, 2006) and sarcomas (Gillette *et al*, 2004; Syed *et al*, 2007), and there are several possible mechanisms by which annexin A2 may be involved in tumour progression. Annexin A2 interacts with tissue-type plasminogen activator and disruption of this interaction resulted in decreased tumour cell invasion (Rand, 2000; Diaz *et al*, 2004; Sharma *et al*, 2006). Annexin A2 is also known to form a complex with cathepsin B that can initiate proteolytic cascades and degrade extracellular matrix proteins. These functions may enhance tumour cell detachment, invasion and motility and thus promote tumour invasion and metastasis (Mai *et al*, 2000). Cell-surface annexin A2 also acts as a receptor for tenascin C, a key extracellular matrix protein involved in epithelial–stromal interactions, and increased annexin A2 expression is associated with progression in pancreatic neoplasia from pancreatic intraepithelial neoplasia through to invasive pancreatic carcinoma (Esposito *et al*, 2006). Recently, it has also been shown that the production of matrix metalloproteinase 1, a key enzyme promoting colorectal cancer invasion (Murray *et al*, 1996), can be mediated by annexin A2. Inhibition of annexin A2 was associated with loss of production of this matrix-degrading enzyme (Zhang *et al*, 2007).

Renal clear cell carcinoma also shows overexpression of annexin A4 and this seems to be related to the metastatic potential of this type of tumour (Zimmermann *et al*, 2004b). Annexin A4 had a distinct subcellular localisation in tumour cells and this was linked to loss of cell-to-cell adhesion and increased tumour cell dissemination (Zimmermann *et al*, 2004b). Additionally, it has been demonstrated that overexpressed annexin A4 promotes cell migration in a model tumour system (Zimmermann *et al*, 2004b), which correlates with our observation that annexin A4 expression increased as tumour stage progressed, such findings are indicative that annexin A4 is implicated in tumour spread. Annexin A4 is known to form complexes with protein kinase C, and there are 10 isoforms of protein kinase C that have roles in cancer progression (metastasis) and some of these isoforms have been shown to be overexpressed in colorectal cancer (Gokmen-Polar *et al*, 2001). It could be through association with protein kinase C isoforms that annexin A4 has an effect on the pathogenesis of colorectal cancer. Annexin A4 has also been shown to be overexpressed in a paclitaxel-resistant cell line and, moreover, overexpression of annexin A4 in this cell line resulted in a four-fold increase in paclitaxel resistance also indicating a role for annexins in anticancer drug resistance (Han *et al*, 2000).

Annexin A11 was overexpressed in colorectal cancer and increased expression correlated with more advanced tumour stage. Annexin A11 is implicated as being involved in cell growth (Farnaes and Ditzel, 2003) and a reduction in annexin A11 expression using RNAi stops cell division (Tomas *et al*, 2004). However, annexin A11 expression was decreased in metastasis, suggesting further dysregulation of this protein with tumour progression and possibly indicating that the tumour microenvironment plays a role in regulating annexin A11, although the specific mechanisms regulating this annexin remain to be elucidated.

Annexin A7 expression was not detected in either normal colon or colorectal cancer, whereas annexin A7 has been proposed as a putative tumour suppressor gene in prostate cancer (Srivastava *et al*, 2001) and that high expression of annexin A7 is associated with poor prognosis in breast cancer (Srivastava *et al*, 2004), thus providing further evidence that there is tumour-type-specific regulation and expression of individual annexins.

In conclusion, this study has shown that annexins A1, A2, A4 and A11 are significantly overexpressed in colorectal cancer and that the overexpression of annexins A2, A4 and A11 showed a significant correlation with increasing tumour stage. The overall expression profile of annexins was associated with survival in colorectal cancer, indicating collectively that annexin expression may contribute to outcome and this would be consistent with the putative roles of annexins in some of the cellular processes that led to tumour invasion. These annexins may also represent tumour biomarkers and potential therapeutic targets (Oh *et al*, 2004; Falsey *et al*, 2006; Wozny *et al*, 2007).

## Figures and Tables

**Table 1:** Clinicopathological data of the patients in this study

**Characteristic**	**Number (%)**
*Gender*	
Male	135 (50.4%)
Female	133 (49.6%)
	
*Age (years)*	
Mean	68
Range	33–92
<70	127 (47.4%)
⩾70	141 (52.6%)
	
*Dukes stage*	
A	53 (19.8%)
B	104 (38.8%)
C	111 (41.4%)
	
*Tumour site*	
Proximal colon	95 (35.4%)
Distal colon	97 (36.2%)
Rectum	76 (28.4%)
	
*Tumour differentiation*	
Well	10 (3.7%)
Moderate	228 (85.1%)
Poor	30 (11.2%)

**Table 2:** Details of annexin antibodies used in this study

**Antibody**	**Source**	**Type**	**Antigen retrieval**	**Dilution**
Annexin A1	BD Bioscience	Monoclonal	Yes	1/100
Annexin A2	BD Bioscience	Monoclonal	Yes	1/100
Annexin A4	Own laboratory	Polyclonal	Yes	1/500
Annexin A7	BD Bioscience	Monoclonal	Yes	1/100
Annexin A11	BD Bioscience	Monoclonal	Yes	1/400

**Table 3:** The relationship of annexin expression and tumour (Dukes) stage

**Annexin**	***χ* ^2^**	***P*-value**	**Interpretation**
A1	5.48	0.484	There is no relationship of annexin A1 expression and tumour stage
A2	23.6	0.001	There is increased expression of annexin A2 with increasing tumour stage
A4	7.1	0.029	There is increased expression of annexin A4 with increasing tumour stage
A7	NA	NA	NA
A11	10.2	0.006	There is increased expression of annexin A11 with increasing tumour stage

NA, not available.

The expression of individual annexins as determined by immunohistochemistry in each tumour stage (Dukes A *vs* Dukes B *vs* Dukes C) was compared to assess the trend in annexin expression (i.e., increase, decrease, no change) with advancing tumour stage.

**Table 4:** Annexin cluster membership

	**Annexin cluster group**			
**Annexin**	**Group 1**	**Group 2**	**Group 3**	**Group 4**
A1	Negative	Negative	Negative	Strong
A2	Negative	Negative	Strong	Weak
A4	Weak	Strong	Moderate	Strong
A7	Negative	Negative	Negative	Negative
A11	Weak	Strong	Strong	Moderate
